# Comprehensive Health Education Combining Hot Spa Bathing and Lifestyle Education in Middle-aged and Elderly Women: One-year Follow-up on Randomized Controlled Trial of Three- and Six-month Interventions

**DOI:** 10.2188/jea.16.35

**Published:** 2005-12-20

**Authors:** Hiroharu Kamioka, Yosikazu Nakamura, Toshiki Yazaki, Kazuo Uebaba, Yoshiteru Mutoh, Shinpei Okada, Mie Takahashi

**Affiliations:** 1 Faculty of Regional Environment Science, Tokyo University of Agriculture.; 2 Department of Public Health, Jichi Medical School.; 3 Japan Health and Research Institute.; 4 International Research Center for Traditional Medicine of Toyama Prefecture.; 5 Department of Physical and Health Education, Graduate School of Education, The University of Tokyo.; 6 Laboratory of Physical Education and Medicine, Mimaki Social Welfare Corporation.

**Keywords:** hot spa, Health Education, Exercise, Middle Age, Aged, Randomized Controlled Trials

## Abstract

**BACKGROUND:**

This study attempted to clarify the duration of effects of 3- and 6-month comprehensive health education programs based on hot spa bathing, lifestyle education and physical exercise for women at 1-year follow-up.

**METHODS:**

We examined middle-aged and elderly women who were randomly divided into two groups and followed up them for one year. Spa programmers instructed subjects for one hour in lifestyle education and physical exercise and for one hour in a half bath (salt spring, temperature at 41.5°C) once a week. The program for the 3-month group (n=19) was repeated in the 6-month group (n=14). The evaluation items were body mass index, PWC75%HRmax (by a bicycle ergometer as aerobic capacity), blood profiles (total cholesterol, HDL cholesterol, arteriosclerotic index, uric acid, and hemoglobin A1c), profile of mood states, self-rating depression scale, subjective happiness, pains in the knee and back, and active modification of lifestyle.

**RESULTS:**

There were significant interactions between groups and response over time to aerobic capacity, hemoglobin A1c, back pain, vigor, fatigue and self-rating depression (respectively, p<0.05). Duration of effects was longer for the 6-month intervention than for the 3-month intervention.

**CONCLUSIONS:**

Beneficial effects of 6-month intervention on hemoglobin A1c, aerobic capacity, pains in the back, vigor, fatigue and depression remained significant at the 1-year follow-up. Duration of effects was longer in the 6-month intervention than in the 3-month intervention.

Japan is one of the countries most abundant in hot spas. An increasing number of people favor bathing in hot spas because an increasing number of hot spa facilities are being built all over Japan.^1^ Combining hot spa bathing in a health education program may prove a useful tool to motivate the active participation of middle-aged and elderly people in the health care projects provided by their local administration.

Hot spas exert a thermal action, an action of hydraulic pressure, a chemical action, and a general conditioning action,^2^ all of which are known to affect humans favorably. Kurabayashi et al. reported that exercise therapy in acid alum springs was effective for the rehabilitation of chronic obstructive pulmonary disease.^3^ Tanizaki et al. reported that underwater exercise in a hot spa pool improved the ventilatory function of patients with steroid-dependent bronchial asthma.^4^^,^^5^ Yokota et al. reported that underwater exercise improved not only asthma symptoms, but also depression and mental conditions.^6^ A recent study by Mitsunobu et al. showed that the effects of spa therapy decreased with increasing levels of bronchial hyperresponsiveness.^7^

Ohtsuka et al. reported that physical exercise in a simple alkaline hot spa pool once or twice (30 minutes) a day for six weeks was effective for improving immunological functions and as a stress-relieving action in patients going through rehabilitation for cerebrovascular disease.^8^ Nobunaga et al. demonstrated that long-term spa therapy over two weeks was not necessarily required for the quality of life (QOL) to improve, but that a short-stay spa therapy (3-7 days) was sufficient for improvement.^9^

Furthermore, some previous studies have attempted to investigate the effects of comprehensive health education programs combined with hot spa bathing. Uehata et al. reported that, as a result of providing guidance for hot spa bathing, lifestyle education, and physical exercise to men of middle and advanced ages, weight decrease, lowered blood pressure, and improved metabolism of serum lipids were observed.^10^ Kamioka et al. reported that two-year program of lifestyle education and physical exercise for the elderly that was centered around underwater exercise in a hot spa pool 15 times a year effectively maintained serum lipid metabolism and mobility, and that the long-term intervention was effective.^11^

One of the tasks of hot spa research is the accumulation of randomized controlled trial (RCT) studies. One report systematically reviewed six studies on spa therapy for rheumatic disease patients in the Cochrane Library, which attaches much importance to the results of RCT.^12^ The reviewer concluded that although the affirmative conclusion of each study could not be ignored, the conclusions should be taken with caution because inadequate methodologies, and the lack of statistical analyses and essential evaluations were found while the accumulation of RCTs is desirable, the favorable results must be reviewed with some skepticism.

Another task of hot spa research is to investigate its effects on healthy subjects. Many studies have reported the therapy results of patients with illness and incidental effects, but few studies have attempted to clarify the effects of hot spa on so-called “relatively healthy people” who have no severe underlying diseases.

In our previous medium-term study with women of middle and advanced ages, an RCT was conducted to compare a control group with a 3-month intervention group meeting once a week for hot spa bathing, lifestyle education and physical exercise. In the results, uric acid, arteriosclerotic index, pains in the back, and psychological tension decreased in the intervention group. The beneficial effects, however, were not sustainable (relapsed) at the 1-year follow-up.^13^ On the other hand, the effects of a 6-month intervention based on the same weekly comprehensive health education program were persistent at the half-year follow-up. It is necessary to further investigate the influence of difference in an intervention period, 3 and 6 months, on the results.

Therefore, this study attempted to verify the duration of the effects on health status of the 6-month intervention at a 1-year follow-up, and to compare the duration of the effects on health status between the 3- and 6-month interventions to clarify the influence of the intervention period length on the duration of effects.

## METHODS

### Subjects

**Figure 1.**
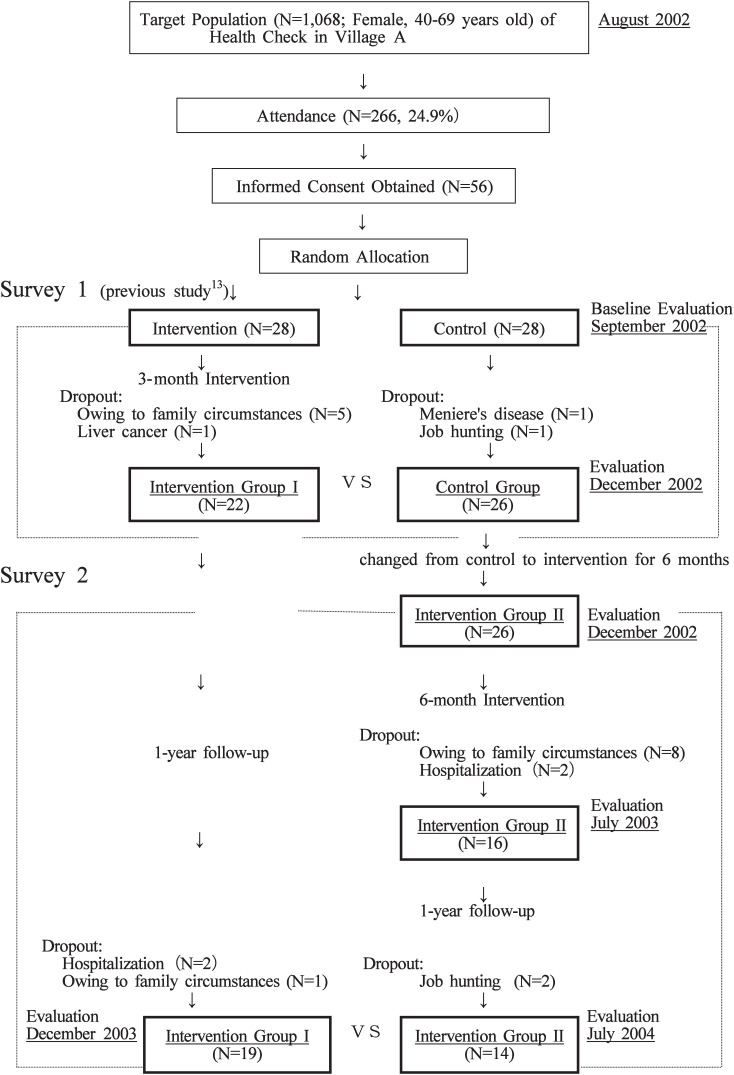
Subject recruitment and research process.

Subject recruiting took place at the periodical health checks (health screening) of Village A in August and September 2002 (Figure 1). Among the 266 women aged from 40 to 69 years who attended the health checks (target population 1068, attendance rate 24.9%), 56 women volunteered for the study. They were randomly divided into an intervention group (Group I) of 28 subjects and a control group (Group II) of 28 subjects. In Survey 1 (3-month intervention), 22 of Group I and 26 of Group II completed the entire program.

After the Survey 1, all the 26 subjects of the Group II were transferred to the 6-month intervention group, and the members of Group I were left for a follow-up without intervention. The Group II was given a 6-month intervention and a follow-up 1 year after the completion of intervention (Survey 2).

The Group II was shifted from a control to an intervention group in the Survey 2 to let every subject participate in the intervention program and receive benefits. Thus, the study design was adopted with maximum consideration of the ethical aspect of the study in cooperation with the health service of the local administration. Written informed consent was obtained from subjects after a thorough explanation of the conditions described above.

### Research Design

This research project was comprised of two designs: (1) verification of the duration of effects on health status of 6-month intervention at 1-year follow-up, and (2) comparison of the duration of effects on health status between the 3-month and 6-month interventions.

### Intervention

In the Survey 1, which has already been reported by Kamioka et al,^13^ a 2-hour program covering hot spa bathing, life style education and physical exercise was given once a week for 12 weeks (Table 1). The subjects participated in half bathing up to the chest in an open-air bath (salt spring, bath temperature at 41.5°C). Bathing time was approximately 20 minutes (2 baths of 10 minutes each), which took approximately 60 minutes, including 40 minutes for changing clothes, washing the body, and rest (drinking beverages). Two spa programmers prepared the bathing program and provided guidance while bathing together with the subjects each time.

**Table 1.** Protocol for bathing, lifestyle education and exercise.

Intervention Group I	
Sessions	Main program (contents)*
--- Introduction and baseline evaluation	
1	A lecture on appropriate bathing method and bathing^†^
2	Stretching, indoor-walking, and bathing
3	Outdoor-walking and bathing
4	A lecture of nutrition and cooking, and bathing
5	Sponge-tennis (short tennis) and bathing
6	A lecture on menopausal syndrome and bathing
7	Underwater exercise in spa pool (1)
8	Prevention exercise for knees and back pain, and bathing
9	Rhythmic exercise and bathing
10	Underwater exercise in spa pool (2)
11	Outdoor-walking and bathing
--- evaluation	
·····1-year follow-up·····	
--- evaluation after 1-year	

	Rates of attendance 9.9 (90.0%) ±1.4times (range:7-11 times)


Intervention Group II	
Sessions	Main program (contents)*

--- Introduction and baseline evaluation	
1	A lecture on appropriate bathing method and bathing
2	Stretching, indoor-walking, and bathing
3	Sponge-tennis (short tennis) and bathing (1)
4	A lecture on nutrition and cooking, and bathing
5	Rhythmic exercise and bathing (1)
6	A lecture on menopausal syndrome, and bathing
7	Outdoor-walking and bathing (1)
8	Underwater exercise in spa pool (1)
9	Prevention exercise for knees and back pain, and bathing
10	Underwater exercise in spa pool (2)
11	Outdoor-walking and bathing (2)
12	Underwater exercise in spa pool (3)
13	Outdoor-walking and bathing (3)
14	Cooking for calorie control, and bathing
15	Underwater exercise in spa pool (3)
16	Rhythmic exercise and bathing (2)
17	Sponge-tennis (short tennis) and bathing (2)
18	Ground golf
19	Rhythmic exercise and bathing (3)
20	Underwater exercise in spa pool (4)
--- evaluation	
·····1-year follow-up·····	
--- evaluation after 1-year	

	Rates of attendance 18.9 (94.5%) ± 1.2 times (range: 16-20 times)

* : Staff members; spa programmer, public health nurse, dietician, exercise instructor, and physical therapist.

† : A salt spring (open-air bath, 41.5°C).

The guidance for lifestyle and physical exercise consisted of lectures (health education) and various types of physical exercise as shown in Table 1. Each session took approximately 60 minutes. Dieticians, public health nurses, physical therapists, and exercise instructors, in addition to the two spa programmers, took part in the lectures and exercise. This 2-hour program was given once a week for 24 weeks in Survey 2 during the period from December 2000 through June 2003. The contents of Table 1 were repeated twice. The method and staff were the same (Table 1).

### Instructions on Daily Life

Guidance on daily life during intervention emphasized increasing physical activity, such as walking instead of driving a car and using stairs instead of using an escalator or an elevator. As for dietary guidance, a leaflet was distributed showing the adequate amount of energy intake studied in the program (Table 1). As for bathing, daily bathing at home or spas was recommended at a suitable water temperature (40-41°C) in a half-bath. Subjects were encouraged to maintain the same attention to lifestyle.

### Examinations and Outcomes

The outcomes of health status were physical indices (height, weight, and body mass index [BMI]), blood profiles (total cholesterol, HDL cholesterol, arteriosclerotic index, uric acid, and hemoglobin A1c [HbA1c]), subjective happiness (Visual Analogue Scale: VAS), severity of pain in the knee and back, and physical working capacity (PWC75%HRmax) by a bicycle ergometer as aerobic capacity. The POMS (Profile of Mood States)^14^^,^^15^ and the Self-rating Depression Scale^16^ were used for the questionnaires on the psychological aspects. The number of active modifications of lifestyle was asked by a questionnaire on the following eleven behaviors: dietary intake, between-meal snacks, sodium intake, weight control, drinking, smoking, working, exercise habits, sleep and rest, stress busters, and dental care. Each item was scored either 1 or 0 (active =1, non active=0), and the total score ranged from 0 to 11. No blind test was used for these measurements.

Blood profiles were examined between 9 to 11 a.m. after fasting longer than 12 hours. Aerobic capacity was measured on a bicycle ergometer (Aero Bike 75XL-II, Combi Corp.), as a ramp test with a continuous increase in load starting from an initial load automatically programmed by sex, age and weight. Physical Work Capacity (PWC75%HRmax) was calculated at 75% of the HRmax estimated from sex and age. For POMS, subjects were asked while in a quiet room to answer frankly about their mood states during the past week.

The methodology (including the protocol and items of survey and measurement) of this study was approved by the Ethical Board of the Laboratory of Physical Education and Medicine, Mimaki Social Welfare Corporation.

### Statistical Analysis

A two-sample *t* test (Welch test) was employed for comparisons between groups with continuous variables in the analysis. Fisher’s exact probability test was performed with discrete variables. A repeated-measures of variance (ANOVA) was used to investigate the differences of change (2 groups × 3 times) between groups. Differences within and among groups were judged significant when significance levels were 5% or less. The SPSS^®^ 11.0J for Windows was used for statistical analysis.

## RESULTS

Table 2 shows the status of underlying diseases. No significant differences were found between the two groups in age, internal diseases, orthopedic diseases, or height (Table 3).

**Table 2.** Clinical characteristics of subjects.

	Intervention Group I	Intervention Group II	
Baseline			
N	28	28	
Age (mean±SD)	59.4±8.6	58.7±7.1	ns
Medical history (Internal medicine)
Hyperlipidemia	6 (21%)	4 (14%)	ns
Hypertension	5 (18%)	7 (25%)	ns
Diabetes	1 (4%)	0 (0%)	ns
Medical history (Orthopedics)
Knee OA	3 (11%)	4 (14%)	ns
Lumbar spine OA	1 (4%)	0 (0%)	ns
Osteoporosis	0 (0%)	1 (4%)	ns

Final evaluation			
N	19	14	
Age (mean±SD)	61.6±7.9	61.4±7.4	ns
Medical history (Internal medicine)
Hyperlipidemia	5 (26%)	2 (14%)	ns
Hypertension	5 (26%)	5 (36%)	ns
Diabetes	1 (5%)	0 (0%)	ns
Medical history (Orthopedics)
Knee OA	3 (16%)	1 (7%)	ns
Lumbar spine OA	1 (5%)	0 (0%)	ns
Osteoporosis	0 (0%)	0 (0%)	ns

ns: not significant, two-sample t test for continuous variables and Fisher’s exact test for categorical variables.

**Table 3.** Effect of intervention on physical characteristics and aerobic working capacity (mean±standard deviation)

Variable	Intervention Group I (n=19)			Intervention Group II (n=14)			Greenhouse- Geisser p value
	
	Baseline	After 3 months	After 1 year follow-up	Baseline	After 6 months	After 1 year follow-up	
Height (cm)	152.4 ± 5.6	152.3 ± 5.6	152.4 ± 5.7	152.5 ± 4.8	152.4 ± 4.8	152.4 ± 4.9	>0.05

Weight (kg)	56.8 ± 7.7	56.3 ± 7.9	57.5 ± 8.4	61.6 ± 10.3	60.3 ± 10.2	60.2 ± 10.1	>0.05

BMI (kg/m^2^)	24.4 ± 2.8	24.2 ± 3.1	24.8 ± 3.2	26.3 ± 3.6	25.7 ± 3.5	26.0 ± 3.3	>0.05

PWC75%HRmax (w)	63.8 ± 17.1	69.8 ± 19.8	66.9 ± 14.3	68.0 ± 16.4	82.5 ± 17.5	81.1 ± 18.8	0.038

Table 4 shows the comparison of baseline values between the subjects who completed the program (completed) and those who dropped out (dropout) in interventions I and II. No significant differences were found in any of the variables. In Group I, PWC75%HRmax increased (from 63.8 ± 17.1W to 69.8 ± 19.8W) by the intervention, but declined again at the 1-year follow-up. In Group II, PWC75%HRmax increased (from 68.0 ± 16.4W to 82.5 ± 17.5W) by the intervention, and remained high (81.1±18.8W) even at the 1-year follow-up (Table 3). There was a significant interaction between groups and response over time in relation to the aerobic working capacity (p<0.05).

**Table 4.** Characteristics of those who completed program and dropouts.

Variable	Intervention Group I			Intervention Group II		
	
	Completed	Dropout		Completed	Dropout	
Number	19	9		14	14	
Height (cm)	152.4±5.6	153.2±5.5	ns	152.5±4.8	153.8 ±5.4	ns
Weight (kg)	56.8 ±7.7	57.3±7.5	ns	61.6±10.3	60.1±7.2	ns
Body mass index (kg/m^2^)	24.4±2.8	24.4±2.7	ns	26.3±3.6	25.4±3.1	ns
PWC75%HRmax (w)	63.8±17.1	61.2±15.5	ns	68.0±16.4	65.8±15.9	ns
Total cholesterol (mg/dL)	213±33	211±29	ns	224±36	217±31	ns
HDL cholesterol (mg/dL)	57.3±11.3	56.8±12.7	ns	56.0±11.8	57.2±10.5	ns
Arteriosclerotic index*	2.86±0.90	2.71±0.92	ns	3.17±1.15	2.79±0.99	ns
Uric acid (mg/dL)	4.43±1.14	4.31±1.05	ns	4.56±0.69	4.34 ±0.88	ns
HbA1c (%)	5.34±0.60	5.29±0.57	ns	5.38±0.29	5.37±0.44	ns
Subjective happiness (%)**	68.7±11.8	67.4±12.2	ns	68.1±17.3	67.6±14.1	ns
Knee pain (%)^†^	17.2±19.8	18.2±17.0	ns	23.3±21.1	21.7±19.8	ns
Back pain (%)^†^	23.5±28.4	21.2±21.0	ns	26.2±20.1	24.6±18.5	ns
Profile of Mood States (POMS: T-score)
Tension	45.3±6.3	47.2±5.9	ns	44.3±5.3	45.0±6.9	ns
Depression	46.3±6.1	46.9±5.7	ns	47.4±4.6	47.1±5.6	ns
Anger	45.1±6.6	47.2±6.9	ns	46.3±5.1	46.8±5.9	ns
Vigor	52.3±10.5	51.2±8.2	ns	55.4±6.3	53.1±8.2	ns
Fatigue	44.2±6.0	45.8 ±6.6	ns	45.2±8.3	46.5±8.9	ns
Confusion	45.9 ±7.5	46.3±6.3	ns	47.3±6.8	47.0±7.6	ns
Self-rating depression scale (pts)	31.8±7.5	32.2±6.9	ns	32.1±6.3	33.0±7.4	ns
Active modification of lifestyle (no.)	4.1±2.1	4.4±2.5	ns	4.7±2.7	4.5±2.1	ns

Value: mean±SD. Two-sample t test (Welch test): ns; not significant.

* : Arteriosclerotic index = (Total cholesterol − HDL cholesterol)/HDL cholesterol

**: 100%: maximal happiness, 0%: maximal unhappiness.

† : 100%: maximal pain, 0%: no pain.

Table 5 shows the results of the blood profiles. In Group II, HbAlc decreased (from 5.38±0.29% to 5.11±0.26%) by the intervention, and remained low at the follow-up (5.17±0.27 mg/dL). There was a significant interaction between groups and response over time to the HbA1c (p<0.05).

**Table 5.** Effect of intervention on blood profile.

Variable	Intervention Group I (n=19)			Intervention Group II (n=14)			Greenhouse- Geisser p value
	
	Baseline	After 3 months	After 1 year follow-up	Baseline	After 6 months	After 1 year follow-up	
Total cholesterol (mg/dL)	213.3 ± 33.3	207.3 ± 30.1	216.4 ± 43.4	223.9 ± 35.6	225.4 ± 32.9	223.2 ± 33.6	>0.05

HDL cholesterol (mg/dL)	57.3 ± 11.3	58.1 ± 11.4	57.6 ± 12.7	56.0 ± 11.8	56.1 ± 10.1	57.5 ± 13.8	>0.05

Arteriosclerotic index	2.86 ± 0.90	2.68 ± 0.83	2.88 ± 0.98	3.17 ± 1.15	3.25 ± 1.16	3.06 ± 1.1	>0.05

Uric acid (mg/dL)	4.43 ± 1.14	4.14 ± 1.12	4.25 ± 1.20	4.56 ± 0.69	4.27 ± 0.76	4.25 ± 0.76	>0.05

Hb_A1c_ (%)	5.34 ± 0.60	5.50 ± 0.58	5.46 ± 0.62	5.38 ± 0.29	5.11 ± 0.26	5.17 ±0.27	0.042

mean ± standard deviation

Arteriosclerotic index = (Total cholesterol − HDL cholesterol) / HDL cholesterol

Table 6 shows the results of subjective happiness and subjective pain in the knee and back. In group II, back pain decreased (from 26.2 ± 20.1% to 17.7 ± 19.2%) at the end of intervention period, and the effect was sustained at the 1-year follow-up (17.7 ± 17.1%). In Group I, pains in the back were alleviated immediately after the intervention (from 23.5 ± 28.4% to 14.2 ± 21.5%), but tended to return to the baseline level one year later. There was a significant interaction between groups and response over time to back pains (p<0.05).

**Table 6.** Effect of rates of subjective happiness and pain (knee and back).

Variable	Intervention Group I (n=19)			Intervention Group II (n=14)			Greenhouse- Geisser p value
	
	Baseline	After 3 months	After 1 year follow-up	Baseline	After 6 months	After 1 year follow-up	
Subjective happiness (%)*	68.7 ± 11.8	71.0 ± 16.0	67.5 ± 18.8	68.1 ± 17.3	69.8 ± 17.2	68.5 ± 18.0	>0.05

Knee pain (%)^†^	17.2 ± 19.8	16.6 ± 25.6	15.6 ± 16.9	23.3 ± 21.1	24.0 ± 25.7	21.2 ± 17.9	>0.05

Back pain (%)^†^	23.5 ± 28.4	14.2 ± 21.5	20.5 ± 27.3	26.2 ± 20.1	17.7 ± 19.2	17.7 ± 17.1	0.025

Tested after root transform of knee and back pain (visual analogue scale)

mean ± standard deviation

* : 100%: maximal happiness, 0%: maximal unhappiness

† : 100%: maximal pain, 0%: no pain

Table 7 shows shifts in mental and psychological status and the number of active modification of lifestyle. There were significant interactions between groups and response over time in terms of vigor, fatigue and self-rating depression (p<0.05).

**Table 7.** Psychological status and active modification of lifestyle.

Variable	Intervention Group I (n=19)			Intervention Group II (n=14)			Greenhouse- Geisser p value
	
	Baseline	After 3 months	After 1 year follow-up	Baseline	After 6 months	After 1 year follow-up	
POMS (T-score)							
- Tension	45.3 ± 6.3	43.2 ± 6.0	47.2 ± 6.9	44.3 ± 5.3	44.9 ± 4.6	43.5 ± 7.7	>0.05
- Depression	46.3 ± 6.1	46.0 ± 5.1	49.8 ± 8.9	47.4 ± 4.6	46.1 ± 5.0	45.6 ± 5.5	>0.05
- Anger	45.1 ± 6.6	44.3 ± 5.5	46.6 ± 6.2	46.3 ± 5.1	44.4 ± 4.0	44.2 ± 5.5	>0.05
- Vigor	52.3 ± 10.5	54.1 ± 8.1	52.7 ± 10.5	55.4 ± 6.3	60.3 ± 8.3	57.8 ± 8.7	0.047
- Fatigue	44.2 ± 6.0	43.5 ± 5.8	47.9 ± 7.6	45.2 ± 8.3	42.6 ± 5.0	42.7 ±4.8	0.035
- Confusion	45.9 ± 7.5	45.2 ± 5.8	49.5 ± 7.8	47.3 ± 6.8	45.4 ± 5.9	46.2 ± 4.6	>0.05

Self-rating depression scale (pts)	31.8 ± 7.5	29.8 ± 5.6	32.1 ± 6.3	32.1 ± 6.3	29.7 ± 6.4	27.9 ± 6.1	0.045

Active modification of lifestyle (no.)	4.1 ±2.1	4.8 ±2.9	4.7 ±2.4	4.7 ±2.7	5.5 ±2.6	5.2 ±2.9	>0.05

mean ± standard deviation

The number of active modification of lifestyle improved both in Groups I and II, but it was not significant.

No subjects complained of pains or sick feelings during the entire program, including the measurement.

Compliance after the interventions was evaluated by exercise habits in daily life. In group II, no one had any particular exercise habits at baseline. Eight (57%) of the 14 subjects established a voluntary exercise club in August 2003, and continue to gather once a week until final evaluation for underwater exercise in spa pool and other various sports. Two (14%) started self-exercise (e.g., walking, exercise in spa pool) once or more per week. The remaining 4 (29%) showed no behavioral change. In Group I, no one had any particular exercise habits at baseline. Eleven (58%) of 19 subjects temporarily joined short-term health classes held by the public administration, but no exercise habit in daily life was observed since then. Two (11%) started walking, and the remaining 6 (31%) were not involved in any exercise.

## DISCUSSION

The subjects were recruited from individuals who underwent health checks in their municipality and were divided randomly into two groups. No significant differences were found between the two groups in any baseline values of age, underlying diseases, physical properties, blood profiles, and mental and psychological status. Therefore, the comparison of the two groups was assumed to be valid as a study design.

Group I of the 3-month intervention yielded good improvements in aerobic capacity, uric acid in serum, pains in the back, and tension immediately after the intervention, but the values tended to return to the baseline level at the 1-year follow-up. This indicates that a 2-hour intervention once a week for three months may not be sufficient.

According to the Transtheoretical model of behavior change (TTM) for exercise,^17^ supposedly, subjects in Group I were at Stage II (contemplation) when they first participated in the program because no one practiced any particular physical exercise prior to the intervention. The reduction of interventional effects during the 1-year follow-up period suggests that the subjects may have reached Stages III (preparation) or IV (action) by the intervention, but not Stage V (maintenance).

In Group II of the 6-month intervention, on the other hand, effects persisted in aerobic capacity, Hb_Alc_, pains in the back, vigor, fatigue and depression even at the 1-year follow-up. The attendance rate was very high (94.5%), in spite of the doubled number of sessions compared to Intervention I. Compliance on physical activity remained high during the following period as more than half of the Group II subjects participated in the voluntary exercise club. These facts exemplify that the most subjects in Group II have reached Stage V (maintenance) in TTM. The formation of a voluntary exercise club may be the result of group dynamics developed during the half-year of communication among participants, and of the supportive advice given by the staff (e.g., recommendations on location and teaching staff). To sustain the effects of intervention, these kinds of social support from the peer and staff members seem to be essential. Short-term intervention, such as once a week for three months, is an easy pattern to conduct as a program by the municipality. However, the above negative results of such a pattern provide valuable suggestions for health administration policies in order to achieve long-term goals. One indication is the necessity for longer intervention to support compliance, e.g., 6 months. Another indication is to add more instruction, for instance, instructions on daily life to modify and establish an active lifestyle. The influence of the frequency of intervention needs to be examined concerning the benefits of more frequent intervention (more than once a week).

The present study was spread over two years from baseline to follow-up. Twelve subjects dropped out in Group II during the process. Because no significant differences were found between subjects who completed the process and the dropouts at baseline, this may have produced a type-II error. However, it is important to determine the reasons for these discontinuations in order for the health administration to examine methods of intervention. The dropouts were asked to tell why they left, if possible, or in a written note by mail if verbal communication was difficult. No subject indicated they dropped out because of dissatisfaction with this study. Their reasons included “emergence of new roles at home to take care of the more elderly or grandchildren,” “getting a job,” “hospitalization for an aggravated underlying disease” and “malignant tumor found.” There may also have been underlying reasons such as difficult human relationships within the group.

The average age at the start of the intervention in Group II was 58.7 ± 7.1 years, which is one of the major transitional periods in life stage, and where preparation for further aging and shifts in present family relationships take place. This result may suggest a problem with long-term intervention (e.g., once a week for longer than six months) that tends to yield better benefits, but is likely to result in more dropouts. The lack of intention-to-treat analysis further limits the discussion.

Back pain was significantly alleviated in the 6-month intervention group in the present study. Strauss-Blasche et al conducted a monthly survey on mood and pains in 268 women who were treated for non-inflammatory chronic pains in the back and arthralgia at a hot spa clinic in Austria.^18^ They reported improved mood and pain relief in spring and autumn, and that temperature, pain, and mood are interrelated. In the present study, both follow-up evaluations made in June and December demonstrated positive effects. Taking into account the low temperature of winter and associated changes (levels of physical activity, appetite, etc.) as confounding factors, the effects of Group II, which underwent intervention during the winter, may be even higher than the indicated values including the outcomes of significant difference (e.g., PWC75%HRmax and Hb_A1c_). Seasonal confounding is one of the potential limitations in the present study.

In the current study, subjects first underwent lifestyle education and physical exercise for 60 min before bathing in an open-air hot salt spring for 60 min including time for changing clothes and washing the body. Relatively vigorous women of middle and advanced ages prefer bathing after physical exercise in general. However, Horikiri et al. reported that elderly people have more improved exercise tolerance after bathing.^19^ From the viewpoint of nursing care and illness prevention, the practice of light physical exercise (e.g., stretching) after bathing may be more appropriate for the somewhat frail elderly. Further research on suitable intervention methods in line with ADL, including timing of bathing, is important for building health with hot spa bathing.

This study concerns the effects of intervention using a combination of hot spa bathing, lifestyle education and physical exercise for women of middle and advanced ages, but there is no control group of hot spa bathing alone. Therefore, the specific effects of the hot spa bathing can not be determined, which in turn limits the scope of discussion. The significant effects of the 6-month intervention, in particular, should be understood as an achievement from the standpoint of comprehensive health education programs that includes utilization of hot spa bathing.

It is assumed that combining health education programs and imposing certain active tasks on participants, as in the present study, is essential. Passive hot spa bathing alone would be difficult as a health policy for municipalities. The accumulation of RCT studies with diverse and realistic designs based on behavioral science is expected to clarify evidence for the effects of hot spa bathing.

The present study was an irregular randomized controlled trial, in which the control group was turned into an intervention group halfway through the study period. However, it was successful in showing that the once-a-week intervention for six months was likely to produce more effects than that same frequency for three months.

In conclusion, the beneficial effects of a 6-month intervention on Hb_A1c_, aerobic capacity, back pain, vigor, fatigue and depression remained significant at 1-year follow-up. Duration of benefits was longer in the 6-month intervention than in the 3-month intervention.
